# 머신러닝을 이용한 한국 지역사회 거주 노인의 낙상 예측 모형 구축: 2차 분석 연구

**DOI:** 10.7586/jkbns.24.024

**Published:** 2024-11-21

**Authors:** Minhee Suh, Hyesil Jung, Juli Kim

**Affiliations:** College of Nursing, Inha University, Incheon, Korea; 인하대학교 간호대학

**Keywords:** Aged, Accidental falls, Supervised machine learning, Secondary data analysis, 노인, 낙상, 머신러닝, 2차 자료 분석

## Abstract

**Purpose:**

This study aimed to develop a fall prediction model for community-dwelling older adults using machine learning.

**Methods:**

The present study was conducted with a secondary data analysis that used data from the 2020 national survey of older Koreans. Among 10,097 participants, data 177 were excluded due to incompleteness and 9,920 were included in the final analysis. Because of data imbalance, upsampling was performed to increase the number of individuals who fell. Forty-five independent variables for fall prediction were selected based on the fall risk factors from previous studies and univariate statistical analysis. The data were split into training and testing sets at an 80:20 ratio. Three machine learning algorithms—logistic regression, random forest, and artificial neural network—were used to develop a fall prediction model.

**Results:**

The random forest model outperformed the others, with an area under the curve of .91, accuracy of .94, precision of .94, recall of .74, and F1 score of .83. An analysis of feature importance revealed that satisfaction with health condition, visual difficulty, instrumental activities of daily living, performance of 400m walk, and cognitive ability were the top five features for fall prediction.

**Conclusion:**

The fall prediction model developed using machine learning demonstrated high model performance, implying its suitability for use as a primary screening tool for fall risk. Subjective satisfaction with one’s health should be considered as an important factor in predicting falls in community-dwelling older adults. It is necessary for community health nurses to reinforce positive health awareness by continuous disease management and physical function improvement for older adults to prevent falls.

## 서론

### 1. 연구의 필요성

노인들에서 낙상 및 낙상 관련 손상은 비교적 흔히 발생하는 안전 사고로, 미국의 경우 지역사회 거주 노인의 약 25%가 낙상을 경험하고 약 10%가 낙상으로 인한 손상을 입는 것으로 알려져 있고[1], 국내 지역사회 노인의 경우 약 15.7%가 낙상을 경험하는 것으로 보고된 바 있다[2]. 노인의 응급실 방문의 가장 많은 원인은 낙상 사고이며, 낙상 사고는 노인 사망의 가장 큰 원인이기도 하다[3]. 뿐만 아니라 손상을 동반하지 않더라도 낙상 사고 경험은 노인들로 하여금 신체활동이나 사회적 활동을 제한하게 할 수 있어[4], 이후 삶의 질에도 부정적인 영향을 미치므로[5], 지역사회 노인들의 낙상을 예방할 수 있는 방안이 필요하다.

효율적인 낙상 예방을 위해서는 낙상 위험요인을 선별하고 낙상을 예측하는 것이 선행되어야 하며, 다양한 낙상 위험요인이 포함되어 예측력이 좋은 낙상 예측 모델을 구축하는 것이 필요하다. 지역사회 노인의 낙상 위험요인으로는 낙상 과거력, 보행 특성, 다양한 질병관련 특성, 약물 복용, 근력, 인지기능, 정신건강 등이 제시되고 있고[6], 다면적 위험요인의 고려가 바람직하다고 알려져 있으나[7], 지역사회 노인들을 위한 낙상 예측 관련 국내 연구로는 낙상 예측 요인 분석에 초점을 맞추어 보고된 연구는 있으나[8,9], 예측 모델의 성능을 평가한 연구는 부족한 실정이다. 그 이유로는 고성능의 낙상 예측 모델을 개발하기 위해서는 낙상 위험요인들을 가능한 모두 포함하여 모델을 구축하는 것이 필요한데, 기존의 통계적 방법으로는 지역사회 거주 노인들의 다양한 낙상 위험요인을 모두 포함하는 모델을 개발하는데 한계가 있기 때문으로 생각된다.

최근 머신러닝 기법이 다양한 영역에서 질병의 위험요인을 선별하고 질병 발생을 예측하는데 활용되고 있다[10]. 머신러닝은 컴퓨터 알고리즘을 이용하여 대단위 자료로부터 위험요인들간의 비선형적 상호작용을 분석하여 예측 모델을 구축할 수 있다. 머신러닝 알고리즘은 자료의 특징을 추출하는 과정에 따라 여러가지 알고리즘이 있는데, 어떤 알고리즘이 유용할지는 자료의 양과 구조에 따라 다르므로 여러 종류의 알고리즘을 분석을 진행하여 도출된 예측 정확도를 보고 선택을 하는 것이 좋은 방법으로 알려져 있다[11]. 머신러닝을 이용하여 지역사회 거주 노인의 낙상 예측 모델을 개발한 국외 연구들을 보면[12-14], 다양한 낙상 위험요인을 포함하여 낙상 예측 모델을 구축하였으나 한 가지 컴퓨터 알고리즘을 사용하여 모델을 개발하였으며, 모델 성능이 area under curve (AUC) 0.65 내외로 우수한 성능을 보였다고 보기에는 어려웠다.

따라서 본 연구에서는 가장 최근에 조사된 노인실태조사 원시자료를 이용하여, 다양한 낙상 위험요인을 분석하고, 여러가지 머신러닝 방법을 적용하여 고성능의 낙상 예측 모델을 구축하고자 한다.

### 2. 연구 목적

본 연구에서는 지역사회에 거주하는 국내 노인의 낙상 관련요인을 다양한 측면에서 분석하고, 다양한 머신러닝 알고리즘을 적용하여 고성능의 낙상 예측 모델을 개발하며, 개발된 예측 모델을 이용하여 낙상에 영향을 미치는 변수 중요도를 확인하고자 한다. 구체적인 연구의 목적은 다음과 같다.

1) 국내 지역사회 거주 노인의 낙상 관련요인을 인구사회학적, 건강상태, 신체기능적, 심리사회적, 환경적 측면에서 분석한다.

2) 머신러닝을 이용하여 국내 지역사회 거주 노인의 낙상을 예측하기 위한 낙상예측 모델을 개발한다.

3) 개발된 예측 모델에 포함된 낙상 예측 변수 중요도를 비교한다.

## 연구 방법

### 1. 연구 설계

본 연구는 2020년 노인실태조사 원시자료를 활용하여 지역사회에 거주하는 국내 노인의 낙상 관련요인을 확인하고, 머신러닝 알고리즘을 적용하여 낙상 예측 모델을 개발하고자 하는 2차 자료 분석 연구이다.

### 2. 연구 대상

연구자료는 한국보건사회연구원에서 공시한 2020년 노인실태조사 원시자료를 제공받아 활용하였다. 노인실태조사는 노인의 생활 현황과 특성 및 욕구를 파악함으로써 노인 복지정책 마련의 기초자료를 제공하고자 시행되고 있다[15]. 연구 대상자는 전국 17개 시ㆍ도의 일반 주거지에 거주하는 65세 이상 노인 10,097명으로, 거주 지역, 연령, 성별, 학력, 유배우 여부 등을 고려하여 조사지역을 선정하며, 선정된 조사구 내 거주하는 모든 노인을 대상으로 노인 본인을 직접 대면하여 조사하였으며, 조사대상 노인이 신체적·정신적인 이유로 응답이 불가능한 경우에는 그 노인과 가장 가까운 가구원이나 가족이 대리 응답하도록 하였다. 자료 수집은 미리 설계된 조사표를 기초로 하여 교육받은 조사원 169명에 의해 2020년 9월 14일~11월 20일 기간 중 수집되었다. 본 연구에서는 불완전하게 응답한 177명의 자료를 제외한 9,920명의 자료를 분석에 이용하였다.

### 3. 연구 도구

#### 1) 종속변수

종속변수는 지난 1년간 낙상 경험 유무이다. 낙상은 ‘귀하께서는 지난 1년간 낙상(넘어짐, 미끄러짐 또는 주저앉음) 경험이 있으십니까?’ 라는 질문에 ‘예, 있습니다’로 응답한 경우를 말한다.

#### 2) 독립변수

독립변수는 지역사회 거주 노인을 대상으로 낙상관련 요인을 분석한 선행연구[2,16]에서 제시되었고, 본 연구에서 단변량 분석 시 낙상 경험 유무와 유의한 관련성을 보였던 변수들로 총 45개를 포함하였다.

##### (1) 인구사회학적 특성

인구사회학적 특성은 나이, 성별, 결혼상태, 교육연한, 독거유무, 현재 경제활동 여부 및 경제상태 만족도를 포함하였다. 경제상태에 대한 만족도는 ‘매우 만족/만족하는 편’은 ‘만족한다’로, ‘그저 그렇다/만족하지 않는 편/전혀 만족하지 않음/잘 모르겠다’는 ‘만족하지 않는다’의 2개 범주로 구분하였다.

##### (2) 건강상태 관련 요인

건강상태 관련 요인으로는 평소 건강상태 및 건강상태에 대한 만족도, 음주 빈도, 시력 및 청력상의 불편함 유무, 다약물 복용, 만성질환 보유 개수, 진단받은 만성질환 종류 및 현재 치료 유무를 포함하였다. 평소 건강상태는 ‘매우 건강하다/건강한 편이다/그저 그렇다/건강이 나쁜 편이다/건강이 매우 나쁘다’의 5개로 분류하여 조사한 원시자료를 그대로 이용하였고, 건강상태에 대한 만족도는 ‘매우 만족/만족하는 편’은 ‘만족한다’로,‘그저 그렇다/만족하지 않는 편/전혀 만족하지 않음/잘 모르겠다’는 ‘만족하지 않는다’의 2개 범주로 구분하였다. 음주 빈도는 ‘전혀 하지 않음/한달에 1회 이하/한달에 2회 이상’으로 구분하였고, 시력 및 청력상의 불편함은 일상생활의 불편함에 대해 ‘매우 불편함/약간 불편함/불편함 없음’으로 원시자료 분류를 그대로 이용하였다. 다약물 복용은 5개 이상의 약물을 복용하는 경우 다약물 복용으로 간주하였고[17], 만성질환 보유 개수는 ‘없음/1-3개/4개 이상’으로 분류하였으며, 만성질환 종류는 단변량 분석에서 낙상경험 유무와 유의한 관련성을 보였던 질환 및 치료 11개 변수를 포함하였다. 치료 여부는 주로 향정신약물 치료를 하는 경우로, 단변량 분석에서 낙상 경험 유무와 유의한 관련성을 보였던, 우울증, 불면증, 양성 전립선 비대 치료 여부를 포함하였다.

##### (3) 신체기능적 요인

신체기능적 요인은 하지근력 제한 여부, 신체적 기능상태, 평소 운동 여부, 일상생활 수행 능력을 포함하였다. 하지 근력 제한 여부는 앉고 일어서기 5회 반복 검사(five times sit to stand test)[18]를 문제없이 수행 가능한 경우와 수행에 제한이 있는 경우로 분류하였고, 신체적 기능상태는 신체기능 척도(physical functioning scale) 중 기동력과 관련된 5개 항목(400m 달리기, 400m 걷기, 휴식없이 10개 계단 오르기, 무릎을 굽히고 앉거나 쪼그려 앉기, 머리 위에 있는 물건 꺼내기, 8kg 이상의 물건 들기)을[19] 측정하여 5개 항목 각각‘문제없이 수행 가능/수행에 제한’으로 구분하였다. 평소 운동 여부는 ‘귀하께서는 평소에 운동을 하십니까?’의 질문에 ‘예/아니오’로 답하도록 한 원시자료를 그대로 이용하였다. 일상생활수행능력(activities of daily living)은 옷 입기, 세수·양치질·머리감기, 목욕 또는 샤워하기, 차려 놓은 음식 먹기, 누웠다 일어나 방 밖으로 나가기, 화장실 출입과 대소변 후 닦고 옷 입기, 대소변 조절하기 등의 7개 항목 모두 독립적 수행이 가능한 경우 ‘완전 독립적’으로, 7개 항목 중 하나라도 독립적으로 수행이 어려운 경우에는 ‘도움이 필요’로 구분하였다. 도구적 일상생활수행능력(instrumental activity of daily livings)은 몸단장, 집안일, 식사준비, 빨래, 제시간에 정해진 양의 약 챙겨먹기, 금전 관리, 근거리 외출하기, 물건 구매 결정·돈 지불·거스름돈 받기, 전화 걸고 받기, 교통수단 이용하기 등의 10개 항목 모두 독립적 수행이 가능한 경우 ‘완전 독립적’으로, 10개 항목 중 하나라도 독립적인 수행이 어려운 경우에는 ‘도움이 필요’로 분류하였다.

##### (4) 심리사회적 요인

심리사회적 요인은 우울상태, 인지기능, 영양섭취 상태를 포함하였다. 우울상태는 노인우울척도 (geriatric depression scale) 15문항을 이용하여 15점 만점으로 평가하였고, 인지기능은 치매선별용 한국어판 간이정신상태검사(Korean version of mini-mental state examination for dementia screening)로 평가하여 30점 만점으로 평가하여 총점을 포함하였다. 영양섭취 상태는 간이영양위험지표 (nutrition screening initiative)를 이용하여 평가하였다. 총 21점 중 0~2점은 양호, 3~5점은 보통, 6점 이상은 불량으로 분류하였다[20].

##### (5) 환경적 요인

환경적 요인은 현재 살고 있는 주택의 종류, 편리한 정도 및 만족도, 지역사회 환경의 전반적인 만족도를 포함하였다. 현재 거주하고 있는 주택의 종류는 ‘단독주택/아파트/연립∙다세대 주택/기타’로 구분하고, 노인이 이용하기에 편리한 정도는 ‘생활하기 불편/불편하지는 않지만 노인 배려 설비 없음/노인배려 설비 있음’으로 구분한 원시자료를 그대로 이용하였고, 현재 살고 있는 주택에 대한 만족도는 ‘매우 만족/만족하는 편’은 ‘만족한다’로,‘그저 그렇다/만족하지 않는 편/전혀 만족하지 않음/잘 모르겠다’는 ‘만족하지 않는다’의 2개 범주로 구분하였다.

### 4. 자료 수집

본 연구는 2020년 노인실태조사 자료를 활용하여 2차 분석한 연구로, 전산으로 코드화된 자료를 이용하여 분석하였다. 원시자료의 자료 수집기간은 2020년 9월 14일~11월 20일이었으며, 본 연구에서 사용한 2차 자료의 분석은 2023년 6월에서 2024년 9월까지의 기간에 진행되었다.

### 5. 자료 분석

본 연구에서 자료분석을 위해 SPSS Statistics ver. 28 for Windows (IBM Corp., Armonk, USA) 통계프로그램 및 머신러닝 라이브러리인 Microsoft azure machine learning studio를 이용하였다. 자료 분석 절차는 아래와 같다.

#### 1) 기초 통계 분석

대상자의 낙상 현황, 인구사회학적 특성 및 건강상태 관련 요인을 파악하기 위해 실수와 백분율, 평균을 산출하였다. 낙상 경험과 관련이 있는 인구사회학적 및 건강상태 관련 요인, 신체기능적 요인, 심리사회적 요인, 환경적 요인을 확인하기 위해 카이제곱 검정 및 t-검정 분석을 시행하였으며, 통계적 유의 수준은 *p* < .05를 기준으로 하였다.

#### 2) 자료준비

선행 연구에서 제시되었던 변수 중 본 연구의 단변량 분석에서 낙상 경험 유무와 유의한 관련성을 보였던 45개의 변수가 독립변수로 선택되었다. 결측치 확인 결과, 건강상태 만족도, 경제상태 만족도, 우울상태, 현재 살고 있는 주택에 대한 만족도 변수에서 177개(1.75%)의 결측치가 있는 것으로 나타났다. 일반적으로 결측치가 5% 미만일 때에는 결측치를 제외하는 것이 추천되므로[21], 결측치를 제외하고 분석하였다.

종속변수의 특정 클래스가 다른 클래스에 비해 극히 낮은 빈도로 발생하여 구성비의 차이가 크게 나는 클래스의 불균형을 해소하여 예측 확률을 높이고자 불균형 자료의 전처리를 하였다. 본 연구에서는 낙상 경험자와 미경험자의 균형을 맞추기 위해서 synthetic minority over-sampling technique를 이용하였다[22]. 선행 연구에 의하면 낙상 경험자와 미경험자의 비율이 1:4일 때 낙상 예측모델 구축에 가장 적절한 것으로 제안되어[23] 낙상 경험자와 미경험자의 수를 1:4로 맞추고자 클래스 빈도가 적은 낙상 경험자의 샘플수를 늘이는 과대표집(upsampling)을 시행하여 모델을 구축하고[24], 그 중 예측 성능이 가장 좋은 모델을 채택하고자 하였다.

#### 3) 머신러닝을 활용한 모델 개발

본 연구에서는 보건의료 분야에서 많이 이용되는 비선형 모델 기반 알고리즘인 로지스틱 회귀분석(logistic regression), 랜덤 포레스트(random forest, RF), 인공신경망 분석(artificial neural network, ANN) 알고리즘을 이용하여 모델을 구축하고, 성능을 평가하였다. 모델 성능의 교차 검증을 위해 전체 자료의 80%를 무작위 추출하여 훈련자료(training set)로 사용하고, 나머지 20%의 자료를 모델 성능 평가를 위한 시험자료(test set)로 활용하였다. 전체 예측 중에서 모델이 올바르게 예측한 비율인 정확도(accuracy), 양성으로 예측된 결과 중 실제로 양성인 자료의 비율인 정밀도(precision), 실제 양성인 자료 중 모델이 양성으로 정확하게 예측한 비율인 재현율(recall), 정밀도와 재현율의 조화 평균인 F1점수(F1 score), 머신러닝 분류 모델 성능을 평가하는 지표인 ROC곡선 아래 면적(AUC)을 산출하여 모델 성능을 평가하였다.

##### (1) 로지스틱 회귀분석(logistic regression)

로지스틱 회귀분석은 다양한 모델을 개발하는데 사용되는 잘 알려진 통계 기법이자 알고리즘으로, 지도 학습 방법의 일종이다. 로지스틱 회귀분석은 모델 구축 결과의 확률을 예측하는 데 흔히 사용되며, 이 알고리즘은 자료를 로지스틱 함수에 맞춰 사건 발생 확률을 예측한다. 로지스틱 회귀분석은 비교적 정확하고 구축과정이 쉬우며, 과적합 가능성이 적고 오차를 최소화하는 관계를 찾는데 우수하여 예측모형을 구축하는데 많이 쓰인다[25].

##### (2) RF

머신러닝에서 랜덤 포레스트 알고리즘은 다수의 의사결정나무를 학습하는 앙상블 학습 방법의 일종으로, 훈련 과정에서 구성한 다수의 결정 트리로부터 부류(분류) 또는 평균 예측치(회귀 분석)를 출력함으로써 예측과 분류 정확도를 개선하고 안정적인 모델을 구축할 수 있다[26]. 랜덤 포레스트 알고리즘은 단일 훈련 자료(training set)로부터 반복적으로 표본을 무작위 복원 추출하여 예측 모델을 구축하고, 그 결과를 평균하는 배깅(bagging) 기법을 사용하여 과적합(overfitting) 문제를 방지한다[23]. 랜덤 포레스트 알고리즘은 모형의 독립변수가 많고 표본의 수가 적은 경우, 예측력이 탁월할 뿐만 아니라 복잡성이 높은 대규모 데이터를 분류하는데도 빠르고 정확한 성능을 발휘하여 최근 대용량 데이터를 분석하는 분야에서 다방면으로 활용되고 있다[27,28].

##### (3) ANN

인공신경망 분석은 딥러닝(deep learning)에 기반한 알고리즘으로, 자료의 통합 및 학습 단계에서 연속된 신경망 모형 층을 구성하여 단계적으로 데이터를 학습하는 기법이다[29]. 이때 신경망 모형 층은 입력층(input layer), 은닉층(hidden layer), 출력층(output layer)으로 구성되어 있으며 입력층에서 전달되는 신호가 은닉층을 거쳐 출력층으로 전달되는 구조이다. 이와 같은 과정은 복잡한 현상에 대해 보다 정확한 분류 및 예측이 가능하다는 장점이 있다[30].

### 6. 윤리적 고려

본 연구는 2차 자료 활용 분석연구로 인하대학교 기관 생명윤리 심의위원회로부터 심의면제를 받았다(IRB 승인번호: 240722-1A). 원자료는 개인정보가 무기명으로 부호화된 형태로 제공받았으며, 분석시에는 암호가 있는 파일 형태로 보관되었다.

## 연구 결과

### 1. 연구 대상자의 인구학적 및 건강상태 관련 요인에 따른 낙상 여부

본 연구에 포함된 대상자는 총 9,920명으로 남성이 3,971명, 여성이 5,949명이었고, 평균 연령은 73.4세였다. 이중 633명이 낙상을 경험하여, 낙상률은 6.4%이었다(Table 1).

대상자의 인구학적 특성 및 건강상태 관련 요인에 따른 낙상 여부의 차이는 Table 1과 같다. 낙상은 연령, 성별, 교육연한, 결혼상태, 독거여부, 주관적 건강상태, 건강상태 만족도, 경제상태 만족도, 직업 유무, 음주 빈도, 시력 및 청력의 불편함, 다약물 복용, 만성질환 개수에 따라 통계적으로 유의한 차이를 보였고, 뇌졸중, 심혈관질환, 관절염, 골다공증, 요통 및 좌골신경통, 골절 후유증, 만성기관지염, 파킨슨병, 불면증, 요실금, 빈혈이 있는 경우와 우울증 치료, 불면증 치료, 양성전립선비대 치료 중인 경우에 유의한 차이를 보였다.

### 2. 대상자의 신체기능적, 심리사회적, 환경적 특성에 따른 낙상 여부

대상자의 낙상과 유의한 관련을 보였던 신체기능적 특성은 하지 근력, 400m 걷기 및 달리기 가능 여부, 쉬지 않고 10개의 계단을 오를 수 있는지 여부, 무릎을 굽히거나 쪼그려 앉기 가능 여부, 팔을 뻗어 머리 위의 물체에 닿을 수 있는지 여부, 8kg의 물건을 들거나 옮길 수 있는지 여부, 일상생활 수행 능력, 도구적 일상생활수행능력, 규칙적 운동 여부였다(Table 2).

심리사회적, 환경적 특성 측면에서 낙상은 우울 정도, 인지기능 정도, 영양섭취 상태에 따라 통계적으로 유의한 차이를 보였고, 환경적 특성 측면에서는 주택의 종류, 현재 거주 주택에 대한 만족도, 지역사회 환경 전반에 대한 만족도에 따라 유의한 차이를 나타냈다.

### 3. 낙상 예측 모델의 성능 평가

로지스틱 회귀분석을 이용한 낙상 예측 모델의 성능은 AUC .76, 정확도 .80, 정밀도 .55, 재현율 .17, F1점수 .26로 정밀도, 재현율 F1 점수가 낮아 우수한 모델로 보기 어려웠다(Table 3). 그러나 랜덤 포레스트 알고리즘을 적용한 낙상 예측 모델은 AUC .91, 정확도 .94, 정밀도 .94, 재현율 .74, F1점수 .83으로 AUC와 F1 점수가 골고루 높아 모델 성능이 가장 우수하였고, 인공신경망 분석을 적용한 모델 역시 AUC .92, 정확도 .90, 정밀도 .79, 재현율 .67, F1점수 .72으로 모델 성능이 우수하였다.

**Figure 1.**
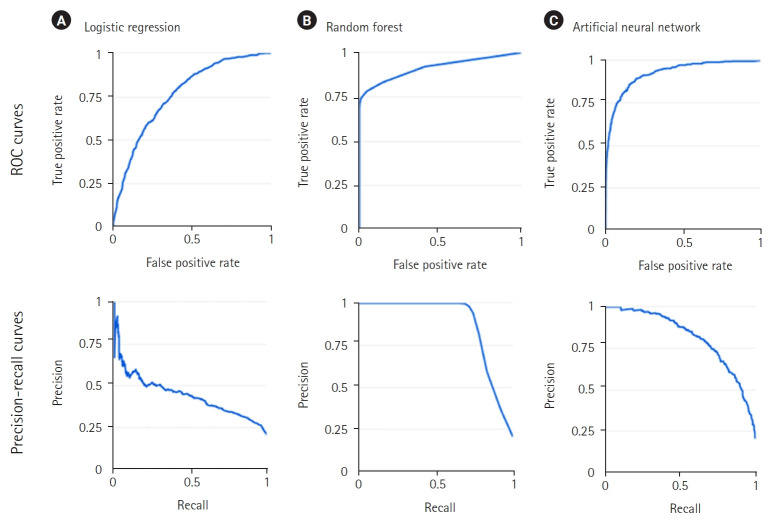
Receiver operating characteristic (ROC) curves and precision-recall curves for each machine learning algorithm.

랜덤 포레스트 및 인공신경망 분석을 적용한 모델의 AUC의 경우 모두 우상단으로 향하는 곡선 모양을 보이고 값 역시 .8이상으로 모델 성능이 좋았고, 정밀도-재현율 곡선 역시 우하향 곡선 모양을 보여 모델 성능이 우수하다고 평가할 수 있었다(Figure 1).

### 4. 낙상에 영향을 미치는 요인

**Figure 2.**
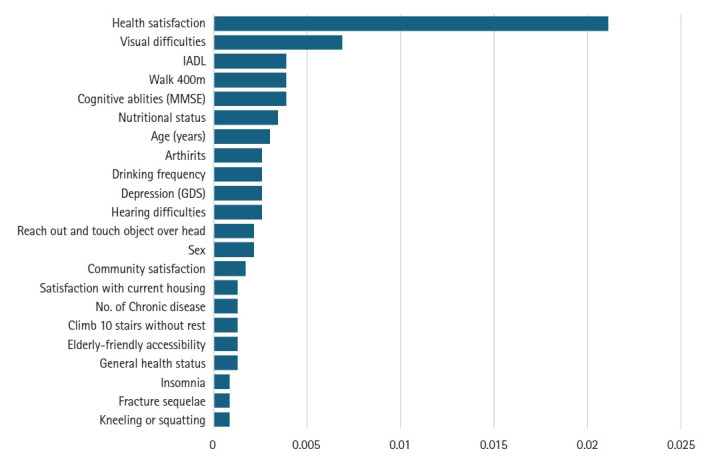
Top 20 features in terms of importance in the fall prediction model from the random forest algorithm. IADL = Instrumental activities of daily living; MMSE = Mini-mental status examination; GDS = Geriatric depression scale.

가장 성능이 좋았던 랜덤 포레스트를 적용하여 개발된 모델로부터 산출한 변수 중요도를 통해 낙상에 기여한 정도가 큰 상위 20개의 요인은 Figure 2에 나타나 있다. 낙상에 기여한 가장 큰 요인은 건강 상태에 대한 만족도였고, 시력 문제, 도구적 일상생활수행능력, 400m 걷기 가능 여부, 인지기능 정도가 상위 5개의 중요 변수였다.

변수 중요도 상위 20개에 포함된 인구학적 요인은 나이와 성별이었고, 건강상태 관련 요인은 건강상태 만족도, 시력문제, 관절염, 음주 빈도, 청력문제, 3개 이상의 만성질환 개수, 주관적 건강 평가, 불면증, 골절 후유증이었다. 신체기능적 요인으로는 400m 걷기 가능 여부, 팔을 뻗어 머리 위의 물체에 닿을 수 있는지 여부, 쉬지 않고 10개의 계단을 오를 수 있는지 여부, 무릎을 굽히거나 쪼그려 앉기 가능 여부, 도구적 일상생활수행능력이었다. 심리사회적 요인으로는 인지기능 정도, 영양섭취 상태, 우울 정도가, 환경적 요인으로는 지역사회 환경에 대한 전반적 만족, 현재 거주 주택에 대한 만족 및 노인이 이용하기에 편리한 정도가 변수 중요도 상위 20개에 포함되었다.

## 논의

본 연구는 지역사회 거주 노인의 낙상 현황 및 관련 요인을 확인하고, 이를 바탕으로 머신러닝을 이용하여 낙상 예측 모델을 개발하고자 하였다. 이를 통해, 국내 지역사회 거주 노인들의 낙상 위험군을 예측하고, 낙상 위험 노인들에게 효율적인 교육 및 중재를 제공하기 위한 기초자료를 제시하고자 한다.

본 연구에서 다양한 머신러닝 알고리즘을 이용하여 낙상 예측모델을 개발하고 평가한 결과, 랜덤 포레스트 및 인공신경망 분석에서 AUC .91~.92, 정확도 .90~.94, 정밀도 .79~.94, 재현율 .67~.74, F1점수 .72~.83으로 전반적으로 성능이 우수한 예측모델을 개발할 수 있는 것으로 나타났으며, 가장 성능이 우수했던 모델은 랜덤 포레스트 알고리즘을 이용하여 개발한 모델로, AUC .91, 정확도 .94, 정밀도 .94, 재현율 .74, F1점수 .83이었다. 이는 선행 연구[13]에서 의사결정나무(decision tree) 알고리즘 머신 러닝을 적용해 개발한 지역사회 노인 낙상 예측 모델의 AUC가 .70이었던 것에 비해 매우 높았는데, 선행 연구의 낙상 예측 모델에서는 낙상 예측 요인을 8~9개 정도만 포함하여 분석하였기 때문에 높은 예측력을 확보하기 어려웠을 것으로 생각된다. 또한 낙상 예측에 있어 의사결정나무 알고리즘 보다는 랜덤 포레스트 알고리즘이 더 적절한 알고리즘으로 볼 수도 있겠는데, Speiser 등[31]이 낙상으로 인한 손상 예측 모델을 개발한 연구에서도 의사결정나무 알고리즘 보다는 랜덤 포레스트 알고리즘을 적용하여 개발한 모델의 AUC가 더 높았다. 뿐만 아니라, 본 연구에서 머신러닝을 이용한 로지스틱 회귀분석을 적용하여 분석한 모델이 성능이 가장 낮고 정밀도-재현율 곡선이 모양이 좋지 않았으며, 특히 재현율이 0.17로 매우 낮아 F1 점수 또한 0.26으로 낮았다. 이는 지역사회 노인의 낙상 모델을 평가한 선행연구[13]에서도 다른 머신러닝 알고리즘에 비해 로지스틱 회귀분석을 적용한 모델의 재현율이 0.50 정도로 가장 낮았던 결과와 유사하였다. 입원 환자의 낙상 예측모델 연구들[32,33]에서도 기존의 통계 분석방법을 이용했던 모델은 성능은 AUC가 .75 내외였던 것에 비해, 머신러닝 방법을 이용하였을 때에는 AUC .95 이상의 고성능의 낙상 예측모델을 개발할 수 있었던 것으로 나타난 바 있다. 따라서 선형 모형을 따르지 않는 다양한 다면적 낙상 위험요인을 모두 포함하여 머신러닝 분석을 하는 경우에는 여러가지 조합을 통해 예측력이 높은 모델을 개발할 수 있는 것으로 보인다. 종합하면, 주기적으로 시행되는 대단위 조사 자료를 바탕으로 머신러닝 기법을 적용했을 때 우수한 낙상 예측이 가능하며, 지역사회에서 노인의 낙상 위험을 사정하고 예측하는 1차 선별 도구로 이용할 수 있음을 시사한다. 추후에 다른 여러 자료를 이용하여 반복 학습을 시켜 모델 적합성과 타당도를 높이는 것이 필요하겠지만, 이러한 과정을 통해 머신러닝 기법을 이용한 낙상 예측 소프트웨어 개발로 이어진다면 지역사회 보건의료 현장에서 이용될 수 있을 것으로 기대할 수 있겠다.

가장 모델 성능이 우수했던 랜덤포레스트 알고리즘을 적용하여 개발한 모델의 예측 요인 중요도를 분석한 결과, 노인의 건강상태에 대한 만족도, 시력 문제, 도구적 일상생활수행능력, 신체적 기능상태 중 400m 걷기 가능 여부, 인지기능 상태가 상위 5개의 중요 예측 요인이었다. 이는 지역사회 거주 노인들의 낙상에는 건강상태 관련 요인, 신체기능적 요인, 심리사회적 요인이 다양하게 관련되는 것으로 볼 수 있으며, 그 중 신체기능적 요인들이 비교적 큰 비중을 차지하는 것으로 볼 수 있겠다. 환경적 요인으로는 지역사회에 대한 만족도 및 주거시설에 대한 만족도가 14, 15번째 중요 예측 요인으로 고려되었으나, 다른 요인들에 비해 상대적으로 중요도가 높지 않았다. 특히 노인 본인이 자신의 건강상태에 만족하는 정도가 가장 중요한 예측 요인이었고, 본인의 건강상태에 대한 부정적 평가 역시 상위 20개 중요 요인에 포함되었다. 이러한 결과는 전혜정과 최주연[34]이 지역사회 노인의 낙상 위험요인을 분석한 결과, 주관적 건강 평가가 가장 큰 요인이었던 점과 유사하였다. 선행 연구들[35,36]에 의하면 노인들이 자신의 건강상태에 대한 주관적 평가나 만족도가 좋지 않으면 낙상 두려움이 증가하고, 이는 활동 저하 및 근력 약화로 이어져 낙상 발생이 증가할 수 있다고 하였는데, 이러한 맥락에서 자신의 건강상태에 대한 불만족은 낙상 위험을 예측하는 주요 요인으로 설명할 수 있겠다. 따라서, 지역사회 노인들의 낙상을 예측함에 있어 노인 본인이 인식하는 건강상태에 대한 주관적인 만족이나 평가를 중요하게 고려할 필요가 있을 것으로 생각된다.

지역사회 거주 노인은 입원 치료가 필요하지 않은 비교적 건강한 노인들이기는 하나, 노인의 특성상 여러가지 만성 질환을 가지고 있고, 각종 약물 치료를 받고 있는 경우가 많은데[37], 본 연구에서도 조사 노인의 83.1%가 1개 이상의 만성질환을 가지고 있었다. 선행 연구들에 의하면[38,39] 당뇨병, 관절염, 뇌졸중, 심부전, 골다공증, 파킨슨병 등의 몇몇 주요 만성질환은 낙상과 관련되는 것으로 보고되고 있다. 본 연구에서는 이러한 질환 외에도 요통 및 좌골신경통, 만성 폐질환, 불면증, 요실금, 빈혈과 같은 지역사회 거주 노인들에게 비교적 흔한 주요 만성 질환들도 단변량 분석에서 낙상과 유의한 관련성이 있는 것으로 나타났고, 결과적으로 총 11개의 관련 질병들이 낙상 예측 모형에 포함되었다. 그러나 예측 요인 중요도 분석 결과, 관절염이 7번째로 중요한 요인이었고, 비록 중요도가 높지는 않았지만 불면증, 골절 후유증이나 만성질환 보유 갯수도 상위 20개 중요 변수에 포함되었다. 지역 사회 노인들을 대상으로 낙상 위험요인을 사정할 때에 그들의 다양한 이상 증상을 확인하거나, 정확한 보행 상태 사정을 하기에는 시간적, 물리적으로 어려운 경우가 많으므로, 이런 경우에는 이들의 만성 질환 정보가 적절히 포함되었을 때 좀 더 우수한 낙상 예측 모형을 개발하는데 도움이 될 것으로 생각된다.

본 연구의 제한점은 다음과 같다. 첫째, 머신 러닝을 적용하여 개발된 낙상 예측 모델의 성능이 우수하였으나, 과적합의 가능성을 배제할 수 없다. 그러나 독립변수 선정시 선행 연구 결과와 단변량 통계 분석 결과를 고려하여 선정하였고, 랜덤 포레스트 알고리즘 적용시 최대 결정 트리 수를 제한하여 과적합의 문제를 최소화하고자 하였다. 둘째, 본 연구는 2차 자료 분석 연구로, 낙상 두려움과 같은 낙상 특이적인 자료나 자세한 약물 복용 정보를 분석에 포함할 수 없었던 점이다. 그러나 Dormosh 등[40]에 의하면 주기적으로 수집된 일반 자료를 통해서도 일정 수준 이상의 낙상 예측 모델을 개발할 수 있다고 하였고, 본 연구에서도 우수한 성능의 낙상 예측 모델을 구축할 수 있었다. 그럼에도 불구하고 수면제, 항우울 약물, 항고혈압 약물 등 만성 질환 치료 약물들이 낙상 발생에 영향을 미칠 수 것으로 알려져 있으므로[41] 추후 연구에서 약물 복용 정보를 통합하여 분석할 수 있다면 좀 더 고성능의 낙상 예측 모델을 개발할 수 있을 것으로 보인다. 셋째로, 본 연구는 머신 러닝을 적용하여 내적 타당성(internal validity)은 평가하였으나, 외적 타당성(external validity)을 평가하지 못하였다. 노인실태조사는 설계된 표본추출방법에 따라 전국 17개 시ㆍ도, 969개 조사구에 거주하는 노인 약 1만명을 대상으로 하나 2020 조사 특성상 코로나-19의 영향으로 특정 조사구의 응답 거부가 발생하여 대표성 확보에 영향을 미쳤으리라 생각된다. 따라서 2023년 노인실태조사 결과를 비롯, 지역사회 노인의 특성을 일반화할 수 있는 대규모의 표본 자료를 이용하여 본 연구에서 개발된 모델의 외적 타당도를 평가할 필요가 있다. 마지막으로, 본 연구에서는 반복 낙상과 초기 낙상을 구분하여 분석하기 어려웠다. 추후에는 반복 낙상과 초기 낙상을 구분하여 분석하는 연구가 필요하다.

## 결론

본 연구에서 2020년 노인실태 조사 자료를 기반으로 다양한 머신러닝 알고리즘을 이용하여 낙상 예측모델을 개발하고 평가한 결과, 랜덤 포레스트 알고리즘을 이용하여 개발한 모델이 AUC .91, 정확도 .94, 정밀도 .94, 재현율 .74, F1점수 .83로 성능이 가장 좋았다. 이러한 결과는 주기적으로 시행되는 대단위 조사 자료를 바탕으로 머신러닝 기법을 적용했을 때 우수한 낙상 예측이 가능하며, 지역사회에서 노인의 낙상 위험을 사정하고 예측하는 1차 선별 도구로 이용할 수 있음을 시사한다. 예측 요인 중요도를 분석한 결과, 노인의 건강상태에 대한 만족도, 시력 문제, 도구적 일상생활수행능력, 신체적 기능상태 중 400m 걷기 가능 여부, 인지기능 상태가 상위 5개의 중요 예측 요인이었으며, 보유 만성질환 중에서는 관절염, 불면증, 골절 후유증의 질환, 그리고 만성질환 개수가 상위 20개 중요 예측 요인에 포함되었다. 그러므로 지역사회 노인들의 낙상을 예측하고 예방하는 데에 있어 신체기능적 요인이나 만성질환 관리 및 이를 기반으로 한 노인의 긍정적 건강상태 인식이 중요할 것으로 생각된다.

본 연구를 통해 다음과 같이 추후 연구를 제언하고자 한다. 첫째, 낙상을 유발하는 것으로 알려진 약물 복용 정보를 포함하여 낙상 예측 모델을 개발하는 연구가 필요하며, 신체기능적 요인 및 만성질환이 낙상 예방에 미치는 영향을 심도있게 조사하는 연구가 필요하다. 둘째 지역사회 노인의 대표성을 확보할 수 있는 다른 자료를 활용하여 낙상 예측 모델의 외적 타당도를 평가하는 연구를 제언한다.

## Figures and Tables

**Table 1.** Demographic and Health-related Factors Associated with Falls (N = 9,920)

Variables	Categories	Total	Fall experience for last 1 year	
			Yes (n = 633)	No (n = 9,287)
Demographic characteristics				
Age (yr)		73.44 ± 6.54	75.26 ± 6.67^***^	73.37 ± 6.51
Sex	Men	3,971(40.0)	191 (30.2)^ ***^	3,780 (40.7)
	Women	5,949 (60.0)	442 (69.8)	5,507 (59.3)
Education (yr)		8.19 ± 4.04	7.12 ± 4.18^***^	8.36 ± 4.03
Marital status	Single	41 (0.4)	5 (0.8)^***^	36 (0.4)
	Married	5,849 (58.9)	267 (42.2)	5,582 (60.1)
	Widowed	3,648 (36.8)	327 (51.7)	3,321 (35.7)
	Divorced	327 (3.3)	31 (4.9)	296 (3.2)
	Separated	55 (0.6)	3 (0.5)	52 (0.6)
Living alone	Yes	3,117 (31.4)	268 (42.3)^***^	2,849 (30.7)
	No	6,803 (68.6)	365 (57.7)	6,438 (69.3)
Financial satisfaction	Satisfied	3,892 (39.2)	155 (24.5)	3,737 (40.2)
	Not satisfied	6,028 (60.8)	478 (75.5)	5,550 (59.8)
Current job	Yes	6,147 (62.0)	453 (71.6)^***^	5,694 (61.3)
	No	3,773 (38.0)	180 (28.4)	3,593 (38.7)
Health-related factors				
General health status	Very healthy	433 (4.4)	12 (1.9)^***^	421 (4.5)
	Quite healthy	4,507 (45.4)	174 (27.5)	4,333 (46.7)
	Average	3,120 (31.5)	196 (30.9)	2,924 (31.5)
	Quite unhealthy	1,659 (16.7)	208 (32.9)	1,451 (15.6)
	Very unhealthy	201 (2.0)	43 (6.8)	158 (1.7)
Health satisfaction	Satisfied	5,212 (52.5)	193 (30.5)	5,019 (54.0)
	Not satisfied	4,708 (47.5)	440 (69.5)	4,268 (46.0)
Drinking frequency	None	6,243 (62.9)	445 (70.3)^***^	5,798 (62.4)
	≤ 1/month	1,389 (14.0)	87 (13.7)	1,302 (14.0)
	≥ 2/month	2,288 (23.1)	101 (16.0)	2,187 (23.6)
Visual difficulties	Very difficult	6,634 (66.9)	326 (51.5)^***^	6,308 (67.9)
	Moderately difficult	3,075 (31.0)	278 (43.9)	2,797 (30.1)
	Not difficult	211 (2.1)	29 (4.6)	182 (2.0)
Hearing difficulties	Very difficult	7,623 (76.8)	429 (67.7)^***^	7,194 (77.5)
	Moderately difficult	2,092 (21.1)	177 (28.0)	1,915 (20.6)
	Not difficult	205 (2.1)	27 (4.3)	178 (1.9)
Polypharmacy	No (< 5)	9,504 (95.8)	562 (88.8)^***^	8,942 (96.3)
	Yes (≥ 5)	416 (4.2)	71 (11.2)	345 (3.7)
Chronic diseases	None	1,678 (16.9)	51 (8.1)^***^	1,627 (17.5)
	1-3	7,082 (71.4)	412 (65.1)	6,670 (71.8)
	≥ 4	1,160 (11.7)	170 (26.8)	990 (10.7)
Type of chronic diseases diagnosed	Stroke	386 (3.9)	45 (7.1)^***^	341 (3.7)
	CAD	449 (4.5)	57 (9.0)^***^	392 (4.2)
	Arthritis	1,570 (15.8)	184 (29.1)^***^	1,386 (14.9)
	Osteoporosis	818 (8.2)	96 (15.2)^***^	722 (7.8)
	Lumbar/sciatic pain	940 (9.5)	119 (18.8)^***^	827 (8.8)
	Fracture sequelae	128 (1.3)	40 (6.3)^***^	88 (0.9)
	Chronic bronchitis,	120 (1.2)	15 (2.4)^**^	105 (1.1)
	Parkinson’s disease	48 (0.5)	10 (1.6)^***^	38 (0.4)
	Insomnia	187 (1.9)	28 (4.4)^***^	159 (1.7)
	Urinary incontinence	306 (3.1)	42 (6.6)^***^	264 (2.8)
	Anemia	131 (1.3)	27 (4.3)^***^	104 (1.1)
Disease under treatment	Depression	120 (1.2)	15 (2.4)^**^	105 (1.1)
	Insomnia	150 (1.5)	25 (3.9)^***^	125 (1.3)
	BPH	325 (3.3)	31 (4.9)^*^	294 (3.2)

Values are presented as the mean ± standard deviation or n (%).

CAD = Coronary artery disease; BPH = Benign prostatic hyperplasia.

*p < .05, **p < .01, ***p < .001.

**Table 2.** Physical, Psychosocial, and Environmental Factors Associated with Falls (N = 9,920)

Variables	Total	Fall experience for last 1 year	
		Yes (n = 633)	No (n = 9,287)
Physical factors			
Muscle power			
Able to perform	7,285 (73.4)	354 (55.9)^***^	6,931 (74.6)
Have difficulty performing	2,635 (26.6)	279 (44.1)	2,356 (25.4)
Run 400m			
Able to perform	1,216 (12.3)	34 (5.4)^***^	1,182 (12.7)
Have difficulty performing	8,704 (87.7)	599 (94.6)	8,105 (87.3)
Walk 400m			
Able to perform	4,900 (49.4)	241 (38.1)^***^	4,659 (50.2)
Have difficulty performing	5,020 (50.6)	392 (61.9)	4,628 (49.8)
Climb 10 stairs without rest			
Able to perform	4,133 (41.7)	140 (22.1)^***^	3,993 (43.0)
Have difficulty performing	5,787 (58.3)	493 (77.9)	5,294 (57.0)
Kneeling or squatting			
Able to perform	4,699 (47.4)	175 (27.6)^***^	4,524 (48.7)
Have difficulty performing	5,221 (52.6)	458 (72.4)	4,763 (51.3)
Reach out and touch object over head			
Able to perform	5,756 (58.0)	287 (45.3)^***^	5,469 (58.9)
Have difficulty performing	4,164 (42.0)	346 (54.7)	3,818 (41.1)
Lift or move objects of 8kg			
Able to perform	4,264 (43.0)	187 (29.5)^***^	4,077 (43.9)
Have difficulty performing	5,656 (57.0)	446 (70.5)	5,210 (56.1)
ADL			
Completely independent	9,499 (95.8)	536 (84.7)^***^	8,963 (96.5)
Help needed	421 (4.2)	97 (15.3)	324 (3.5)
IADL			
Completely independent	8,918 (89.9)	470 (74.2)^***^	8,448 (91.0)
Help needed	1,002 (10.1)	163 (25.8)	839 (9.0)
Regular exercise			
Yes	5,187 (52.3)	306 (48.3)^*^	4,881 (52.6)
No	4,733 (47.7)	327 (51.6)	4,406 (47.4)
Psychosocial factors			
Depression (GDS)	4.71 ± 2.28	5.01 ± 2.35^***^	4.67 ± 2.37
Cognitive abilities (MMSE)	24.32 ± 5.34	23.48 ± 5.85^***^	24.34 ± 5.33
Nutritional intake status			
Good	7,210 (72.7)	324 (51.2)^***^	6,886 (74.1)
Normal	1,857 (18.7)	196 (31.0)	1,661 (17.9)
Poor	853 (8.6)	113 (17.8)	740 (8.0)
Environmental factors			
Type of current housing			
Detached house	3,931 (39.6)	215 (34.0)^***^	3,716 (40.0)
Apartment	4,700 (47.4)	303 (47.9)	4,397 (47.3)
Townhouse	1,205 (12.2)	104 (16.4)	1,101 (11.9)
Others	84 (0.8)	11 (1.7)	73 (0.8)
Elderly-friendly accessibility			
Inconvenient	975 (9.8)	72 (11.3)	903 (9.7)
Not inconvenient but not elderly-friendly	7,078 (71.4)	460 (72.7)	6,618 (71.3)
Not elderly-friendly	1,867 (18.8)	101 (16.0)	1,766 (19.0)
Satisfaction with current housing			
Satisfied	7,460 (75.2)	443 (70.0)^**^	7,017 (75.6)
Not satisfied	2,460 (24.8)	190 (30.0)	2,270 (24.4)
Community satisfaction			
Satisfied	6,357 (64.1)	362 (57.2)^***^	5,995 (64.6)
Not satisfied	3,563 (35.9)	271 (42.8)	3,292 (35.4)

Values are presented as the mean ± standard deviation or n (%).

ADL = Activities of daily living; IADL = Instrumental activities of daily living; GDS = Geriatric depression scale; MMSE = Mini-mental status examination.

*p < .05, **p < .01, ***p < .001.

**Table 3.** Model Performance According to Machine Learning Algorithms (N = 9,920)

Algorithms	Accuracy	Precision	Recall	F1 score	AUC
Logistic regression	.80	.55	.17	.26	.76
Random forest	.94	.94	.74	.83	.91
Neural network	.90	.79	.67	.72	.92

AUC = Area under the curve.

## Data Availability

The National Survey of Older Koreans data are available on request through the website, Health and welfare data portal (https://data.kihasa.re.kr/kihasa/main.html).
